# Personalized Medicine in Ophthalmology: From Pharmacogenetic Biomarkers to Therapeutic and Dosage Optimization

**DOI:** 10.3390/jpm3010040

**Published:** 2013-03-05

**Authors:** Frank S. Ong, Jane Z. Kuo, Wei-Chi Wu, Ching-Yu Cheng, Wendell-Lamar B. Blackwell, Brian L. Taylor, Wayne W. Grody, Jerome I. Rotter, Chi-Chun Lai, Tien Y. Wong

**Affiliations:** 1 Illumina Inc., San Diego, CA 92122, USA; 2 Department of Biomedical Sciences, Cedars-Sinai Medical Center, Los Angeles, CA 90048, USA; 3 Department of Ophthalmology, Chang Gung Memorial Hospital and College of Medicine, Chang Gung University, Taoyuan 333, Taiwan; 4 Singapore Eye Research Institute, Singapore National Eye Centre, 168751, Singapore; 5 Department of Ophthalmology, Yong Loo Lin School of Medicine, National University of Singapore and National University Health System, 119074, Singapore; 6 Departments of Pathology and Laboratory Medicine, Pediatrics and Human Genetics, David Geffen School of Medicine, University of California, Los Angeles, CA 90095, USA; 7 Department of Medicine, Cedars-Sinai Medical Center, Los Angeles, CA 90048, USA; 8 Department of Pediatrics and Human Genetics, David Geffen School of Medicine, University of California, Los Angeles, CA 90095, USA

**Keywords:** personalized medicine, pharmacogenetics, clinical utility, ophthalmology, VEGF, age-related macular degeneration, glaucoma, retinopathy, drug delivery, nanotechnology

## Abstract

Rapid progress in genomics and nanotechnology continue to advance our approach to patient care, from diagnosis and prognosis, to targeting and personalization of therapeutics. However, the clinical application of molecular diagnostics in ophthalmology has been limited even though there have been demonstrations of disease risk and pharmacogenetic associations. There is a high clinical need for therapeutic personalization and dosage optimization in ophthalmology and may be the focus of individualized medicine in this specialty. In several retinal conditions, such as age-related macular degeneration, diabetic macular edema, retinal vein occlusion and pre-threshold retinopathy of prematurity, anti-vascular endothelial growth factor therapeutics have resulted in enhanced outcomes. In glaucoma, recent advances in cytoskeletal agents and prostaglandin molecules that affect outflow and remodel the trabecular meshwork have demonstrated improved intraocular pressure control. Application of recent developments in nanoemulsion and polymeric micelle for targeted delivery and drug release are models of dosage optimization, increasing efficacy and improving outcomes in these major eye diseases.

## 1. Introduction

Reported seven years ago, the first demonstrated success of genome-wide association studies (GWAS) was the discovery of association between Y402 allele polymorphism in the complement factor H (*CFH*) gene and a 7.4-fold increased likelihood of developing age-related macular degeneration (AMD) [1]. This finding spawned a revolution in genetics research, with GWAS eventually demonstrating association for approximately 250 traits in over 1,700 publications to date [2] for diseases ranging from inflammatory bowel disease to coronary artery disease [3]. There was immense potential that these studies may lead to clinical utility via discovering variants manifested in the prediction of disease risk, but these genotypic-phenotypic associations may also predict response to therapy. However, while the association between pharmacogenetic biomarkers and personalized medicine has proven invaluable in some areas of medicine, such as oncology [4], the clinical application of pharmacogenetic biomarkers faces challenges in others [5]. In ophthalmology, the clinical utility of pharmacogenetic biomarkers is debatable. The polygenic etiology of ophthalmic diseases, compounded by multi-factorial environmental/lifestyle contributions to disease development and progression, such as age, gender, diet and smoking, all have to be considered when discussing the clinical utility and added value of genetic testing.

Aside from pharmacogenetics, another means of personalized tailoring of therapeutics in ophthalmology is in therapeutic and dosage personalization. For AMD, one of the most common causes of visual loss in elderly people, prior to the introduction of anti-vascular epithelial growth factor (VEGF) therapies, thermal laser photocoagulation or photodynamic therapy (PDT) with verteporfin were the preferred modalities for neovascular AMD, However, the regimen was highly dependent on the disease type and the location of the abnormal vascular leakage on fluorescein angiography [6]. The recent approval of a fusion protein that binds to all VEGF-A isoforms, as well as placental growth factor, has shown fewer required injections, which translates to fewer risk of iatrogenic complications [7,8]. Alternative therapies in the form of dietary supplements, minerals and antioxidants may also be useful in AMD. For other conditions, such as glaucoma, although several risk factors for glaucoma progression have been identified, the reduction of intraocular pressure (IOP) remains the only proven strategy to delay glaucoma progression. Newly synthesized prostaglandin analogs and several new drugs in the novel category of Rho-kinase inhibitors that act on the trabecular meshwork are currently being developed. In other retinal disease, such as diabetic macular edema (DME), retinal vein occlusion (RVO) and retinopathy of prematurity (ROP), laser, the only available treatment previously, effectively halts the progression of disease in the vast majority of patients; however, these treatments frequently destroy a large portion of the retina [9,10]. Anti-VEGF therapies are of high clinical utility and can decrease the need for laser treatment or vitreoretinal surgery. Nanotechnology bodes to be very promising in delivering personalized therapeutics to the eyes with non-invasive modalities that are preferable over surgery. Nanoemulsion and polymeric micelles have been shown to be efficacious and superior in reducing adverse outcomes associated with intravitreal injections. There is also the potential of sustained-release of drugs and personalized targeting with monotherapy or combination therapy.

## 2. Pharmacogenetic Biomarkers

### 2.1. Age-Related Macular Degeneration

Age-related macular degeneration (AMD) is the most common cause of visual impairment in the elderly and is classified as either exudative (wet) or non-exudative (dry) in its later stages [11]. Ninety percent of severe vision loss is caused by the exudative form of AMD [12]. There is some evidence that the two anti-VEGF therapeutics used to treat AMD, ranibizumab (Lucentis; Genentech Inc. South San Francisco, CA and Novartis International AG, Basel, Switzerland) and bevacizumab (Avastin; Genentech Inc. South San Francisco, CA, USA), have differing responses based upon the individual patient’s genotype (Table 1) [13,14]. Bevacizumab is a humanized anti-VEGF monoclonal antibody [15] first used successfully as an anti-angiogenic agent in metastatic colorectal cancer. It has also been used with good outcomes in treating many retinopathies with VEGF up-regulation, including AMD [16,17], diabetic retinopathy [18,19,20], vitreous hemorrhage [21,22], neovascular glaucoma [23], pathological myopia [24] and retinal vascular occlusion [25,26,27]. Ranibizumab, an anti-angiogenic agent approved to treat exudative AMD, is a monoclonal antibody fragment derived from the same parent mouse antibody as bevacizumab with stronger affinity for binding to VEGF-A receptor. The therapeutic and dosage personalization of these drugs are discussed in greater detail in subsequent sections.

In the case of intravitreal bevacizumab, *CFH* Y402H genotypes, TC and TT, show more than five-fold increased improvement compared to the CC genotype [28]. However, there was no statistically significant difference in the response to bevacizumab with the LOC387715 (*ARMS2*) genotype, which along with the high temperature requirement of A1 (*HTRA1*), is strongly associated with increased risk of AMD [28,29]. The data shows that after treatment with bevacizumab, visual acuity of the patients improved from 20/248 to 20/166 (TT) and from 20/206 to 20/170 (TC), but actually decreased from 20/206 to 20/341 for the CC genotype (*p* = 0.016) [28]. In a prospective study with twice the number of patients, the CC genotype was confirmed to have worse outcome as measured by distance and reading visual acuity [30]. In a similar experiment with intravitreal ranibizumab, the TC and TT genotypes for *CFH* showed improvement with fewer injections compared to the CC genotype [13]. Over a nine-month period, patients with the CC genotypes received one additional injection (*p* = 0.09). Recurrent analysis showed that patients homozygous for the *CFH* Y402H risk allele (CC) were 37% more likely to require additional ranibizumab injections (*p* = 0.04) [13]. Another study found that individuals homozygous for 69S in *ARMS2* had decreased central subfield retinal thickness and no improvement in visual outcomes compared to improved visual acuity in *ARMS2* rs10490924 and rs1061170 genotypes following ranibizumab treatment [31].

**Table 1:** Pharmacogenetic biomarkers for age-related macular degeneration (AMD) and glaucoma.

Disease	Drug	Gene	Variant	Clinical Outcome
AMD	Bevacizumab	*ARMS2*	LOC387715	No difference in visual acuity
		*CFH*	Y402H (TT and TC)	More than five-fold improvement in visual acuity
		*CFH*	Y402H (CC)	Worse outcome for distance and reading visual acuity
	Ranibizumab	*ARMS2*	69S Homozygotes	Decrease in central subfield retinal thickness; no improvement in visual acuity
		*ARMS2*	rs10490924, rs1061170	Improved visual acuity
		*CFH*	Y402H (TC and TT)	Fewer injections needed
	Photodynamic therapy (PDT)	*CFH*	Y402H	No difference in PDT treatment
		*CRP*	rs2808635, rs877538	Increased response to PDT
		*MTHFR*	C677T	Increased response to PDT
		*PT*	G20210A	Increased response to PDT
		*VEGF*	rs699947, rs2146323	Decreased response to PDT
Glaucoma	Prednisolone acetate	*GR*	N363S	Steroid-induced ocular hypertension
	Triamcinolone acetonide	*GR*	BcII, N766N and within intron 4	No correlation with magnitude of intraocular pressure elevation
	Beta-adrenergic blockers (topical)	*ADRB2*	rs1042714	Increased response (Intraocular pressure reduction of 20% or more)
	Timolol (topical)	*CYP2D6*	R296C (TT and CT)	More likely to develop bradycardia
		*CYP2D6*	R296C (CC)	Less likely to develop bradycardia
	Latanoprost (0.005% topical)	*PR*	rs3753380, rs3766355	Increased response (Intraocular pressure reduction of 15% or more)

Gene abbreviations: *ADRB2*, Adrenergic receptor beta-2; *ARMS2*, Age-related maculopathy susceptibility protein 2; *CFH*, Complement factor H; *CRP*, C-reactive protein; *MTHFR*, Methylenetetrahydrofolate reductase; *PR*, Prostaglandin F receptor (2 alpha); *PT*, Prothrombin; *GR*, Glucocorticoid receptor; *VEGF*, Vascular endothelial growth factor. PDT: photodynamic therapy.

The *CFH* Y402H genotype showed no association with the effectiveness of photodynamic therapy (PDT) [32,33], another treatment option detailed below. On the other hand, there was a significant association found between the effectiveness of PDT and two C-reactive protein (*CRP*) single nucleotide polymorphisms (SNPs) with homozygous alleles GG at rs2808635 (GG; OR = 3.92; 95% CI (1.40–10.97); *p* = 0.048) and AA at rs877538 (AA; OR = 6.49, 95% CI (1.65–25.47); *p* = 0.048) [33]. Another significant determinant of the effectiveness of PDT was found in the *VEGF* gene [34]. For rs699947, the allele frequency for AA, AC and CC genotypes were 14%, 39% and 46% in PDT non-responders compared to 40%, 48% and 12% in PDT responders, respectively (*p* = 0.0008). For rs2146323, the frequency for AA, AC and CC genotypes were 4%, 32% and 64% in non-responders and 24%, 38% and 38% in responders, respectively (*p* = 0.0369) [34]. Furthermore, associations were observed between methylenetetrahydrofolate reductase (*MTHFR* C677T) and prothrombin (*PT* G20210A) polymorphisms with PDT effectiveness [35]. In 96 patients, PDT responders were more likely to have the mutations *MTHFR* C677T (OR = 6.9; 95% CI (2.7–18.1); *p* < 0.001) and *PT* G20210A (OR = 5.6; 95% CI (1.2, 27.2); *p* = 0.03).

These data suggest that knowing the patient’s genotype could allow for individualization and optimization in dosage and treatment. However, one cannot overlook environmental contributors to the development of AMD, such as smoking and body mass index (BMI) [36,37,38]. Taking genetics and environmental factors together, the *CFH* Y402H homozygous CC genotype with BMI ≥ 30 and smoking conferred the greatest risk [39]. Age, gender and other factors also have a complementary impact and thus further limiting the efficacy, reliability and application of pharmacogenetics in the treatment of AMD. Finally, many of the above studies are also limited by their retrospective study design, inconsistent re-treatment criteria and small sample sizes [40].

### 2.2. Glaucoma

Glaucoma is the leading cause of irreversible blindness worldwide, estimated to affect 70 million and causing blindness in about 10% of these affected individuals [41]. The precise mechanism responsible for this progressive neurodegenerative damage to the axon of the optic nerve has yet to be fully elucidated so the standard of care is to treat the elevated IOP. The therapeutic and dosage personalization of glaucoma therapeutics are discussed in greater detail in subsequent sections. As in AMD, there are several examples of differing therapeutic responses based on individual genotypes in glaucoma (Table 1). Glucocorticoid administration has been found to elevate IOP in some patients, causing them to develop steroid-induced glaucoma. Those with a glucocorticoid receptor variant type N363S were found to have a positive correlation to prednisolone administration and elevated IOP [42]. The lack of a statistically significant relationship was observed in patients with another glucocorticoid receptor polymorphism, N766N, where intravitreal triamcinolone acetonide injection had no effect on IOP elevation [43]. Furthermore, a differing efficacy in the therapeutic lowering of IOP by beta-blockers was observed for patients with a CC genotype coding at androgenic receptor beta-2 (*ADRB2*) [44]. Additionally a similar IOP lowering effect for topical latanoprost, a prostaglandin analogue, was found to correlate to two SNPs in the prostaglandin receptor [45]. In terms of side effects, the *CYP2D6*/R296C polymorphism was associated with the development of bradycardia in some patients with topical timolol treatment [46]. Patients with the TT and CT genotypes developed bradycardia (*p* = 0.009), while patients with the CC genotype seemed to be resistant [46]. There are also racial differences in response to timolol and beta-blockers. Two studies show differing degrees of effectiveness when ethnicity was considered, but both showed less overall efficacy in African American than in Caucasian patients [47,48]. The etiologies of racial differences are currently being studied for a variety of disorders, but its application to ophthalmology and the understanding of its mechanisms are largely still unknown [49].

Currently, the clinical utility of pharmacogenetics in glaucoma may be low; however, the application of pharmacogenetics may have the potential to determine the most effective class of drug to lower IOP and the proper dosage for each individual patient based on genotype. The selection of candidate genes to study some of the relevant pathways that have yet been sufficiently delineated could facilitate narrowing the list of possible targets. However, even if there were polymorphisms identified, the expression of these polymorphisms may introduce yet another variable into the system as evidenced by the cross-influence of pathways within target ocular tissue, such as the ciliary body [50]. 

## 3. Therapeutic Personalization

### 3.1. Age-Related Macular Degeneration

Age-related macular degeneration (AMD) is the leading cause of visual impairment in the elderly. In its advanced stages, it is classified as either geographic atrophy (dry AMD) or choroidal neovascularization (wet AMD) and is associated with significant irreversible blindness [6,51]. Dry AMD accounts for 90% of all cases and is characterized by accumulation of drusen, leading to progressive atrophy of the retinal pigment epithelium (RPE), choriocapillaris and photoreceptors. At present, no definite treatment is available for geographic atrophy, though dietary supplements with lutein and zeaxanthin have been shown to be strongly associated with reducing AMD risk [52]. High-dose antioxidants and minerals may also delay the progression from intermediate to advanced AMD, as was found in the Age-Related Eye Disease Study (AREDS) [53]. The original AREDS dietary formula contains β-carotene, which has been shown to cause lung cancer in both current and past smokers. Thus, in the era of personalized medicine, a modified formula—removal of β-carotene, addition of lutein and zeaxanthin and reduction of zinc—in the AREDS-2 is currently being developed [54].

Though dry AMD accounts for a majority of the cases, wet AMD, characterized by immediate visual loss with rapid progression, is responsible for 90% of severe visual loss. The hallmark of wet AMD is neovascularization originating from the choroid plexus, extending into the subretinal space, leaking blood and fluids, eventually causing fibrous scarring and ultimately resulting in permanent damage to central vision. Before the advent of anti-VEGF therapy for ocular conditions, thermal laser photocoagulation or PDT with verteporfin were the preferred modalities for neovascular AMD, but the regimen was highly dependent on the type (classic, occult or mixed) and the location (subfoveal, juxtafoveal or extrafoveal) of the abnormal vascular leakage on fluorescein angiography (Table 2). 

The Macular Photocoagulation Study (MPS) found a significant decrease of visual deterioration in subjects with extrafoveal or juxtafoveal lesions treated with laser photocoagulation [55], but was less effective in patients with subfoveal lesions [56], as it caused iatrogenic central scotoma. Yet, despite somewhat promising results with laser photocoagulation, persistent or recurrent choroid neovascularization (CNV) was seen in about half of the patients after a five-year follow-up [57]. Treatment then evolved to PDT with verteporfin, which was mainly indicated for subfoveal CNV. This involves an intravenous injection of verteporfin, a photosensitizing dye that preferentially concentrates at the pathological choroidal tissue, followed by activation with light of a specific wavelength. This process creates oxygen-free radicals that cause a direct occlusion of the pathological vasculature, while preserving normal tissues. Results from the Treatment of AMD with PDT (TAP) and the Verteporfin in Photodynamic Therapy (VIP) studies show that vision remained stable in a majority of patients with classic CNV at two-year follow-up, but was less beneficial in patients with occult CNV [58]. A subsequent study found that lesion size was an important prognostic factor in PDT treatment, irrespective of lesion type [59].

**Table 2:** Management of ophthalmic angiogenic disorders.

Ocular Intervention	Neovascular AMD	DME	BRVO	CRVO	ROP
**Macular focal/grid laser photocoagulation**	Recommended for extrafoveal or juxtafoveal lesions.	Recommended for DME and should be initiated 6 weeks before PRP.	Recommended for macular edema and VA ≤ 20/40 (not recommended if macular ischemia is present).	Not recommended for treatment of macular edema due to CRVO.	_
**Scatter/pan-retinal laser photocoagulation**	_	_	Recommended for retinal or disc neovascularizations.	Recommended for anterior-segment neovascularization. Not recommended if without neovascularization, unless follow-up every 4 weeks is not possible.	Recommended for type 1 ROP
**Photodynamic therapy with verteporfin**	Indicated for subfoveal lesions prior to anti-VEGF era. Less beneficial in occult CNV.	_	_	_	_
	Recommended for PCV, either alone or as combination therapy with anti-VEGF agents.				
	Effective in RAP as combination therapy.				
**Intravitreal triamcinolone acetonide injections**	Effective in RAP as combination therapy.	Recommended for DME. Contraindicated in advanced glaucoma and steroid responders.	Not superior to macular grid laser photocoagulation in improving VA and associated with a higher adverse outcome.	Improvement in VA given 1mg every 4 months compared to observation.	_
**Intravitreal dexamethasone implants**	_	Phase 3 clinical trial underway	Improvement in VA given 0.7 mg every 6 months compared to sham implants. Contraindicated in advanced glaucoma or steroid responders.	Improvement in VA given 0.7 mg every 6 months compared to sham implants. Contraindicated in advanced glaucoma or steroid responders.	_
**Intravitreal anti-VEGF injections**	Recommended as first line of therapy for subfoveal lesions. Ex: Pegaptanib, ranibizumab, bevacizumab and aflibercept.	Current data supports the use of anti-VEGF agents for DME.	Improvement in VA with monthly 0.5 mg ranibizumab for 6 months follow by as needed basis compared to sham/ 0.5 mg ranibizumab injections after 2 years of follow-up. Treatments with 1.25 mg bevacizumab show promising outcome in small case series.	Improvement in VA with monthly 0.5 mg ranibizumab for 6 months follow by as needed basis compared to sham/0.5 mg ranibizumab injections after 2 years of follow-up. Treatment personalization (follow-up interval and dosage) is recommended in the second year of treatment. Treatments with 1.25 mg bevacizumab show promising outcome in small case series.	Intravitreal 0.625 mg bevacizumab was beneficial for zone I, but not zone II stage 3+ ROP compared to laser photocoagulation Systemic safety still under investigation.
	Less effective in PCV as monotherapy. Requires combination therapy with PDT.				
	Effective in RAP as combination therapy.				

Abbreviations: AMD, age-related macular degeneration; BRVO, branch retinal vein occlusion; CNV, choroidal neovascularization; CRVO, central retinal vein occlusion; DME, diabetic macular edema; PCV, polypoidal choroidal vasculopathy; PDT, photodynamic therapy; PRP, pan-retinal photocoagulation; RAP, retinal angiomatous proliferation; ROP, retinopathy of prematurity; VA, visual acuity; VEGF, vascular endothelial growth factor.

The most recent advance in the treatment of wet AMD was the introduction of anti-VEGF therapies, currently regarded as the standard of care. Pegaptanib (Macugen, Pfizer), a drug that specifically targets the VEGF-165 isoform, was effective for AMD and first received US FDA approval in 2004 [60]. Subsequently, the second US FDA-approved anti-VEGF therapy for AMD was ranibizumab (Lucentis, Genentech/Novartis), a recombinant, fragmented, monoclonal antibody that binds to all VEGF isoforms. The MARINA study compared ranibizumab (0.3 mg or 0.5 mg) against sham injections in subjects with minimally classic or purely occult CNV. Over a two-year period, over 90% of either treatment group had visual stabilization (loss of <15 letters) compared to 53% in the placebo group. More importantly, 34% of subjects who received the 0.5 mg dose had visual improvement that was maintained for over two years, demonstrating for the first time that a treatment for AMD had significant visual gain [61]. The ANCHOR study compared monthly intravitreal ranibizumab injections (0.3 mg or 0.5 mg) *versus* PDT for predominantly classic CNV. In this study, 94.3% and 96.4% of subjects who received the 0.3 mg and 0.5 mg ranibizumab respectively lost fewer than 15 letters, compared to 64.3% of subjects who received PDT [62]. These two landmark studies demonstrated that intravitreal ranibizumab was not only superior to sham or PDT therapy for the treatment in neovascular AMD, but resulted in significant visual improvement, altering the treatment paradigm for neovascular AMD. 

Though not US FDA-approved for ocular treatment, bevacizumab (Avastin, Genentech), a recombinant full-length monoclonal antibody that also binds to all VEGF isoforms, is commonly used as an off-label treatment in ocular angiogenic disorders, since it has similar functions as ranibizumab, but is much lower in cost. The CATT trial found that bevacizumab had similar efficacy as ranibizumab administered either on a monthly basis or as needed. Visual improvement was similar in both treatment groups. There was slightly less visual improvement in subjects treated on an as-needed basis (an average of 10 fewer injections in a two-year period) compared to those who received monthly injections. There was also a higher rate of systemic adverse events in the group treated with the unlicensed bevacizumab [63]. Recently approved by the US FDA for the treatment of neovascular AMD, aflibercept (Eylea, Regeneron/Bayer), a fusion protein that binds to all VEGF-A isoforms, as well as placental growth factor, is another key player. It was introduced as a newer therapeutic agent that requires fewer injections compared to other anti-VEGF therapies. Aflibercept given 0.5 mg monthly, 2 mg monthly or 2 mg bimonthly after an initial loading dose demonstrated similar efficacy, compared to monthly injections of 0.5 mg ranibizumab. Of particular interest, the regimen of 2 mg bimonthly injections after three monthly loading doses required fewer injections, which translates to fewer risk of endophthalmitis [7]. Furthermore, visual acuity was maintained for one year after an initial three monthly loading dose, followed by subsequent as-needed dosing schedule [64].

There is increasing evidence that Asian patients with neovascular AMD have a variant of AMD, termed polypoidal choroidal vasculopathy (PCV), which is characterized by polypoidal lesion with inner choroidal vessel abnormality. PCV is more prevalent in Asian subjects, accounting for about 50% of neovascular AMD compared to 10% in Europeans [65]. Studies have shown that PCV does not respond as well to anti-VEGF therapies as compared to PDT [65], and combination therapy with PDT and ranibizumab was associated with a more favorable outcome compared to ranibizumab monotherapy [66,67]. Current treatment for PCV remains undefined and given the growing number of neovascular AMD patients in Asia, new clinical trials are clearly needed that specifically investigates AMD in Asian populations [68]. Another variant of AMD in the spectrum of occult CNV is retinal angiomatous proliferation (RAP), which represents about 12%–15% of neovascular AMD [69]. It is associated with proliferation of intraretinal capillaries with retinal anastomosis or CNV. Current treatment for RAP is unclear, though a combination of laser, triamcinolone, PDT and anti-VEGF therapy show some benefits [70,71].

Ocular complications with intravitreal anti-VEGF therapeutics are usually mild, including pre-retinal or vitreous hemorrhage (1%), cataract (1%) and exotropia (1%). Additional potential benefits of anti-VEGF therapy compared to ablative therapies include simplicity of procedure, elapsed time for the procedure, savings on equipment for alternative therapies, such as laser or cryotherapy, less destruction of the retina, improved follow-up with regression of tunica vasculosa lentis and dilation of pupils and the elimination of complications associated with ablative treatments, such as refractive errors or visual field loss [72,73,74]. No systemic complications, such as neuro-developmental delay, stroke, heart attack, myocardial infarction or vaso-occlusive disorder have been reported thus far. However, studies in both animals [75,76] and human beings [77,78] have shown that minor fractions of anti-VEGF therapeutics circulate into the systemic circulation.

### 3.2. Diabetic Macular Edema

Diabetic retinopathy (DR), a frequent complication of diabetes, is the leading cause of preventable blindness in working-age adults [9]. DR is clinically classified as non-proliferative DR and proliferative DR. Diabetic macular edema (DME), the most common cause of visual loss in subjects with diabetes, is a separate classification assessed independently from the DR spectrum, because it can develop at any stage of DR. The pathogenesis of DR and DME is thought to be related to the loss of pericytes, thickening of basement membrane and endothelial cell loss, leading to microaneurysms, blood-retinal barrier breakdown, increase in inflammation and vascular leakage. There are several treatment modalities for DME (Table 2). The goal of laser treatment is to reduce disease progression by targeting areas of leakage on the retina. The Early Treatment Diabetic Retinopathy Study (ETDRS) was the first study to examine laser photocoagulation in the treatment of DME [79]. It was shown in this study that focal/grid laser photocoagulation reduced the risk of moderate visual loss by 50% in subjects with DME. Intravitreal triamcinolone acetonide (IVTA) has also been shown to significantly reduce DME, with maximal action at one week and lasting 3–6 months [80,81]. IVTA can be used as primary therapy or in conjunction with laser photocoagulation [82]. Focal/grid laser photocoagulation has also been studied in conjunction with IVTA and the combination has been found to be more effective with fewer adverse side effects than IVTA for DME over a 24-month period [83].

As with AMD, recent advances in anti-VEGF therapeutics have contributed much to the evolution of treatment for DME [84]. In a phase II prospective clinical trial, pegaptanib sodium appeared to improve visual outcome in DME patients [85]. In the READ-2 (phase II randomized multi-center) trial ranibizumab was shown to significantly improve visual acuity at month six compared to laser [86]. In the phase III RESTORE trial, ranibizumab monotherapy was shown to improve visual acuity (+6.1 letters) compared to laser alone (+0.8 letters) or even ranibizumab with laser (+5.9 letters) [87]. Ranibizumab was approved in August 2012 by the U.S. FDA for DME, primarily based on phase III trials RIDE and RISE [88]. At 24 months, 34% of the patients in ranibizumab 0.3 mg treated group (*vs*. 12% in the control group) in RIDE and 45% of the treated patients (*vs*. 18% in the control group) in RISE were able to read at least three additional lines or 15 letters. The average gains exceeding two lines (10 letters) in both treated groups in RIDE and RISE were significantly higher than in the control group at 24 months. In just one week after the first treatment, there was significant gain in average vision for the treated groups, and the vision improvements observed at 24 months were maintained with continued treatment through 36 months. Similar to AMD, bevacizumab has also been studied in small pilots and efficacy has been documented. The Diabetic Retinopathy Clinical Research Network conducted a phase II prospective randomized multi-center trial and concluded that intravitreal bevacizumab (IVB) can reduce DME [85]. The efficacy of repeated IVB with laser treatment has also been evaluated by the Bevacizumab or Laser Therapy in the Management of Diabetic Macular Edema (BOLT) study. At 12 months, the laser group lost an average of 0.5 ETDRS letters, while the bevacizumab group gained eight ETDRS letters [89], and this improvement was maintained at 24 months [90]. 

### 3.3. Retinal-Vein Occlusion

Retinal-vein occlusion (RVO) is the most common retinal vascular disorder after diabetic retinopathy in the elderly and is often associated with systemic disorders, such as hypertension, hyperlipidemia, diabetes mellitus and/or arteriosclerotic vascular disorders [10,91]. It is classified as branch retinal vein occlusion (BRVO) or central retinal vein occlusion (CRVO), depending on the site of occlusion and further divided into ischemic (non-perfusion) or non-ischemic (perfusion) RVO, each with differing prognosis and treatment. Macular edema and retinal neovascularization are the two most common causes of visual impairments [92]; thus, ocular managements with laser photocoagulation, intravitreal injections of glucocorticoids or anti-VEGF agents, as well as other surgical or systemic therapies, have focused on these two sequelae (Table 2).

There are two types of laser photocoagulations used in the treatment of RVO. Macular grid laser photocoagulation is mainly indicated for the treatment of macular edema, and scatter (pan-retinal) laser photocoagulation is indicated for the prevention and treatment of retinal and/or disc neovascularization. The Branch Vein Occlusion Study [93] showed improvement by two or more lines from baseline in 65% of eyes treated with grid photocoagulation, compared to 37% in untreated eyes after a three-year follow-up. Grid laser photocoagulation is thus indicated for visual acuity (VA) ≤20/40 and poor vision, due to macular edema in BRVO without macular ischemia. Conversely, in the Central Vein Occlusion Study, VA, did not improve in eyes with macular edema treated with grid laser photocoagulation compared to untreated eyes (VA 20/200 *vs.* 20/160, respectively) after a three-year follow-up [94]. Both the Branch and Central Vein Occlusion Studies indicate use of scatter laser photocoagulation when neovascularization is present [95,96]. It is not recommended as a prophylactic treatment in ischemic CRVO when neovascularization is not present [96].

Intravitreal injections of triamcinolone acetonide have been used to treat macular edema in several other ocular etiologies, due to its potent anti-inflammatory properties. Though the exact mechanism is unknown, it is believed that triamcinolone acts by reducing VEGF concentration in the vitreous, leading to a reduced capillary permeability, resolving macular edema and, consequently, improving VA. Case series have reported decrease of macular edema and visual improvement with the use of intravitreal triamcinolone in RVO [97,98,99]. However, a large randomized trial does not support the use of intravitreal injection of triamcinolone for macular edema in BRVO. The Standard Care *versus* Corticosteroid for Retinal Vein Occlusion (SCORE) Study compared the visual outcome of macular grid photocoagulation with 1 mg or 4 mg of intravitreal triamcinolone treatment in eyes with macular edema due to BRVO [100]. Visual acuity was similar in all three groups after a one-year follow-up. However, adverse outcomes, such as elevated IOP and cataract formation, were much more frequent in subjects treated with triamcinolone injections compared to laser treatment, and this observation was dose-dependent [100]. Conversely, results from the SCORE-CRVO trial showed that intravitreal triamcinolone was associated with significant VA improvement compared to the standard therapy of observation over a 12-month period. Adverse outcome was dose-dependent; thus current guideline recommends the use of 1 mg dose in the treatment of macular edema secondary to CRVO [101]. 

Ranibizumab is regarded as the most frequent anti-VEGF agents used in the treatment of RVO [102,103,104,105]. Two large multi-center studies, the Ranibizumab for the Treatment of Macular Edema following Branch Retinal Vein Occlusion (BRAVO) [104,106] and Ranibizumab for the Treatment of Macular Edema after Central Vein Occlusion Study (CRUISE) [107,108] examined the efficacy and safety of intravitreal injection of ranibizumab in the treatment of macular edema secondary to BRVO or CRVO, respectively. In both studies, eyes were randomized to monthly sham, 0.3 mg ranibizumab or 0.5 mg ranibizumab injections in the first six months [104,107]. Following this, treatments were offered on an as-needed basis of the assigned ranibizumab dosage in the treatment group, and the sham group was assigned to 0.5 mg ranibizumab after the sixth month. In BRAVO, VA improved an average of 12.1, 16.4 and 18.3 letters in the sham, 0.3 mg and 0.5 mg ranibizumab treatment groups, respectively, after one year of follow-up [106]. Similarly results were seen in CRUISE after a one-year follow-up [108]. In both BRAVO and CRUISE, the sham group gained additional VA following ranibizumab injections, but observable improvement at the twelfth month was not similar to the extent of that seen in the ranibizumab groups, suggesting that early intervention (timing) with ranibizumab is a critical factor in the determinant of favorable visual outcome in macular edema for both BRVO [106] and CRVO [108]. 

In the 13–24 month period, approximately 85% of subjects in BRAVO and 87% of subjects in CRUISE were enrolled in HORIZON, a follow-up study during the second year of treatment where subjects were evaluated every three months and re-injected with 0.5 mg ranibizumab for recurrent macular edema. During this study, the US FDA approved ranibizumab for the treatment of RVO and the protocols were terminated. As a result, the variability of follow-up periods in subjects with BRVO and CRVO show that visual outcome in BRVO patients remained stable even with decreased injections and follow-up time, but subjects with CRVO were greatly affected. A reduction in the treatment frequency was associated with loss of benefit in CRVO patients. Thus, subjects with CRVO require treatment individualization, indicating that both the follow-up intervals and number of injections should be personalized in patients with CRVO in the second year of treatment [109]. More recently, preliminary results from small case series and short-term follow-up show promising results of intravitreal bevacizumab in the treatment of RVO. Optimal dose determination and injection intervals/frequency are currently being investigated [25,26,110,111,112]. Treatment for associated systemic disorders, such as hypertension, hyperlipidemia, diabetes mellitus and/or arteriosclerotic vascular disorders, should also be performed in concert to ocular treatments.

### 3.4. Retinopathy of Prematurity

ROP remains one of the leading causes of childhood blindness. In late stages of ROP, neovascularization of abnormal or pathological vessels arise, due to retinal immaturity, and lead to retinal traction, detachment, hemorrhage and funnel configuration, eventually resulting in poor vision. Neovascularization is mainly driven by VEGF [113], and currently, the recommended treatment for Type-1 ROP is peripheral ablation by laser. The timing of treatment has moved to earlier stages of the disease, as a result of the Early Treatment for Retinopathy of Prematurity Study (ETROP) [114]. Although laser effectively halts the progression of stage 3 ROP to stage 4 ROP in 90% of patients, these treatments frequently destroys approximately two-thirds of the retina. Furthermore, some patients progress to retinal detachment despite laser or cryotherapy. The functional outcomes are still not satisfying in stage 4B or stage 5 ROP, even after vitrectomy or scleral buckling [115,116,117]. Hence, a new treatment that could either decrease the need for laser treatment or vitreoretinal surgery would be of high clinical utility (Table 2).

Since VEGF is highly elevated in advanced ROP and has been found to play a central role as the driving force for neovascularization [118,119,120], the blocking of VEGF by anti-VEGF agents is a logical approach. Mintz-Hittner *et al.* showed that a single injection of bevacizumab prevented progression to retinal detachment in eyes with posterior zone I ROP even without laser ablation [121]. Their recent randomized trial of BEAT-ROP [122] showed that intravitreal bevacizumab (IVB) monotherapy, as compared to conventional laser therapy in infants with Stage 3+ retinopathy of prematurity, showed a significant benefit for zone I, but not zone II disease. Development of peripheral retinal vessels continued after treatment with IVB, However, conventional laser therapy led to permanent destruction of the peripheral retina [122]. The results are encouraging, because roughly 27% to 47% of posterior zone 1 cases progress to retinal detachment, even with the application of peripheral retinal ablation [123,124,125]. Wu *et al.* also found similar results in a multi-center study in Taiwan with 27 patients (49 eyes) [126]. The neovascularization regressed after IVB (0.625 mg) monotherapy and resulted in retinal full vascularization [122] (to zone 3) in roughly 90% of eyes with pre-threshold ROP, either as a primary treatment or a salvage treatment after laser therapy. The other 10% of eyes needed additional laser, repeated injection of bevacizumab or vitrectomy, either because of none response to IVB or worsening of ROP after IVB [126].

Although limited, most of the studies to date of bevacizumab use in ROP show positive response [121,127,128,129,130,131,132,133,134,135,136,137,138]. Additional long-term studies will be needed to replicate these findings [139]. There is ongoing concern regarding the systemic safety of IVB in newborns, because of the lack of supportive data either in large animals or humans [140]. Among the studies using IVB for ROP, the BEAT-ROP study is the only prospective randomized study. Even though it showed the efficacy of IVB, the study does not address systemic safety issues, because of insufficient sample size. Other studies found lowered systemic VEGF for up to two weeks after 1 mg or 0.5 mg of IVB use in ROP patients showed no evidence of systemic adverse events [78]. A pathological study of the eyes of a very low-birth weight infant (350 g) born at 22 weeks gestational age showed no local toxic effects to the retina with continued retinal differentiation and vascularization following two injections of intravitreal bevacizumab (0.50 mg in 0.02 mL solution) [130]. Therefore, the systemic safety of IVB and a standard treatment guideline for ROP remain inconclusive [141,142]. Other growth factors, such as insulin-like growth factor 1 (IGF-1) may also play a role in the pathogenesis of ROP [75,118,119,120,143,144] and warrants further investigation.

### 3.5. Glaucoma

Although several risk factors for glaucoma progression have been identified, the reduction of IOP remains the only proven strategy to delay disease progression. Current IOP-lowering medications include beta-blockers, prostaglandin analogs, alpha-adrenergic agonists, carbonic anhydrase inhibitors or a combination of these drugs, achieving IOP reduction by increasing aqueous humor outflow or decreasing aqueous humor production. Based on the results of meta-analyses of randomized clinical trials, prostaglandin analogs are the most effective in reducing IOP, achieving 27% to 33% reduction from baseline in patients with open angle glaucoma or ocular hypertension [145,146]. Even though the exact mechanism is not fully understood, currently, it is thought that prostaglandin analogs act by stimulating the activity of matrix metalloproteinases and relaxing the ciliary muscle, leading to widening spaces between muscle bundles and, thus, increasing uveoscleral outflow [147,148,149]. Prostaglandin analogs are progressively replacing beta-blockers as first-line medical therapy owing to their efficacy, lack of relevant systemic side effects and need for fewer instillations. At present, commercially available derivatives of prostaglandin, including latanoprost, travoprost, isopropyl unoprostone, bimatoprost and tafluprost, all have similar efficacy. Glaucoma patients are usually on topical therapies with at least one drug for decades; therefore, toxic effects from preservatives, such as benzalkonium chloride (BAK), in anti-glaucoma medications are of concern. Long-term exposure to preservatives in anti-glaucoma medications results in deleterious effects on ocular surface, such as conjunctival hyperemia, cellular apoptosis and inflammatory cell infiltration of the conjunctiva [150,151,152]; thus, preservative-free IOP-lowering formulations have been developed to reduce ocular-surface side effects.

Tafluprost is a newly synthesized prostaglandin analog, with high affinity for the fluoro- prostaglandin receptor [153,154]. It is being developed in both preservative-containing and preservative-free formulations. A multi-center phase III study showed that tafluprost had a substantial IOP-lowering effect, with a mean decrease in diurnal IOP of 7.1 mmHg from baseline, which is similar to the effect of 7.7 mmHg for latanoprost [155]. The IOP-lowering effect of preservative-free tafluprost was not inferior to that of preservative-free timolol [156]. Furthermore, preservative-free tafluprost showed less toxicity in human conjunctival epithelial cell lines, compared to preserved prostaglandin analogs, such as latanoprost, travoprost and bimatoprost [157]. Likewise, travoprost was recently developed into two formulations preserved with poliquaternium-1 and sofZia, which are less toxic and better tolerated than BAK [158]. The metrics of glaucoma treatments are shifting from maximal efficacy to increased patient adherence and ocular surface protection [159]. These preservative-free prostaglandin analogs may have great potential of higher patient adherence to treatment if compared with the other preservative- containing prostaglandin analogs, in particular in patients with co-existing ocular surface diseases.

As the main common cause of IOP elevation in open angle glaucoma is the decrease of aqueous outflow facility through the trabecular meshwork, several new drugs that act on the trabecular meshwork are currently investigated. This has opened a new horizon for a novel class of IOP-lowering medications. For example, Rho-kinase (ROCK) inhibitors have been shown to remodel trabecular actin cytoskeleton in animal models and human cell cultures, thus increasing aqueous drainage through the trabecular meshwork [160,161]. Although no derivatives of ROCK inhibitors are currently on the market, at least two have entered early clinical trials [159]. Latrunculin-B (Lat-B), an actin cytoskeletal disruptor that decreases IOP by decreasing the resistance to aqueous humor outflow through trabecular meshwork [162,163,164], is currently in clinical trials as a novel anti-glaucoma drug. When treated with Lat-B, scanning electron microscopy showed 2.5-fold more pores in the inner wall of Schlemm’s canal and a 64% increased in the outflow facility of aqueous humor [163].

## 4. Nanotechnology for Dosage Optimization

The delivery of effective therapeutics for eye disorders has undergone important advances in recent years. Recent developments in nanomedicine, including nanoemulsion and polymeric micelles, have presented novel technologies for eye therapeutics that reduce toxicities, sustain drug delivery and reduce the number of treatments. These new delivery systems offer advantages over previous modalities in that they are non-invasive and preferable to surgery. The issues for effective therapeutics in the case of retinopathy are to deliver and penetrate the globe with active drug molecules and sustained release of these drugs to retinal tissues in therapeutic concentrations. In glaucoma, the main concerns are reducing IOP and increased survival of photoreceptors and retinal ganglion cells. The most common therapeutic delivery mechanisms are systemic drug delivery, topical administration and intravitreal injection [165]. Systemic drug delivery allows drugs to reach regions of the eye both via oral and intravenous administration. In patients with cytomegalovirus infection for example, delivery of the drug valganciclovir systemically resulted in less unwanted side effects [166]. An obvious inherent problem of systemic delivery system is the increased off-target effects and the increased toxicity. Also, drugs may be modified before reaching its intended target and modulation of drug concentration must be considered when comparing therapeutic benefit to damage, due to uptake in other tissues [167].

Besides systemic delivery, topical administration in the form of eye drops can also be used to deliver drugs to the eye and has its greatest success when the targeted region of the eye is easy to reach. Specifically, anterior eye abnormalities are routinely treated with this delivery system [168]. SAR 1118 delivered by ophthalmic drop has been shown to last up to eight hours and reduce the blood-retinal barrier breakdown associated with diabetic retinopathy [169]. However, eye drops have not been effective to treat eye aliments in which there are physiological barriers compounded by the tear circulation [170]. In addition to decreased effective access to the posterior eye and requiring multiple administrations [169], topical drugs may cause more cell death, because of increased drug administration or length of exposure, as in the case of ethacrynic acid [171]. Compared to topical administration, intravitreal injection is advantageous, due to its ability to bypass barriers, and allows for direct drug administration to affected regions. However, this method also has the disadvantage of requiring multiple applications [167]. In recent years, the introduction of a potent, bio-degradable dexamethasone intravitreal implant (OZURDEX, Allergan, Irvine, California) have shown promising results for the treatment of macular edema secondary to BRVO or CRVO [172]. The micronized dexamethasone is gradually released by a drug-copolymer complex and sustains the concentration over several months. In a six-month trial, subjects receiving the dexamethasone implant demonstrated improvement in VA and a faster recovery period compared to sham injections in macular edema following BRVO or CRVO, though adverse outcomes, such as increased IOP and cataract formation, should be carefully monitored [172]. A phase III trial for DME using Posurdex biodegradable implant (sustained release of dexamethasone) is also under way. Another steroid implant (fluocinolone acetonide, Retisert) has shown good results with patients with DME, but its adverse effect profile is concerning with a majority of patients developing cataracts within 36 months [173]. A phase III trial for fluocinolone is also under way to evaluate the Alimera injectable implant.

Recent advances in drug delivery for AMD and glaucoma rest upon improvements in polymeric micelles and nanoemulsion. These technologies improve the packaging of therapeutics for more efficacious treatment and are able to delivery all drugs produced on the nanoscale, including proteins, DNA and peptides. These technologies use liposomes or polymers to package and protect drugs en route to regions of the eye [174]. Not only are they advantageous because both lipophilic and lipophobic drugs can be solubilized in these emulsions, nanoemulsions have increased stability and can reach deeper eye regions [175]. Poly(fumaric anhydride) and poly(lactide-co-glycolide) can be mixed to make polymeric micelles, which have been shown to deliver active drugs to different targets in the body [176]. These polymers can be modified to increase specificity and improve delivery of drugs to targeted regions [177]. Moreover, packaging of drugs into polymeric micelles have been shown to have decreased toxicity [178], and biodegradable versions of polymeric micelles further limit toxicities [179].

As discussed previously, anti-VEGF drugs are the most common drugs used in the treatment of AMD [180], and recent studies utilizing nanoemulsion and polymeric micelle delivery of these drugs continue to show improved clinical utility. Polymeric micelles containing the anti-VEGF drug EYE001 and bevacizumab both resulted in sustained delivery eye for AMD treatment [181,182]. Moreover, choroidal neovascularization associated with AMD can be treated well by micelle packaged pDNA [183]. In glaucoma, liposome delivery of latanoprost has been shown to be stable and increased sustained delivery in comparison to topical administration of the drug [184]. Open-angle glaucoma can also be treated by delivering brimonidine, encapsulated in nanoemulsions, to achieve long sustainability and lower IOP *in vivo* [185]. Studies have shown that liposomes that are neutral in charge have improved sustainability [186]. Moreover, polymeric micelles composed of dendrimer hydrogel polymers can delivery both brimonidine and timolol maleate to various regions of the eye [187]. Interestingly, these drugs delivered together are more effective than when delivered individually via this platform [187]. Nanoemulsion formulations have also been shown to provide improved drug delivery of glial cell line-derived neurotrophic factor (GDNF) to the retinal ganglion cells (RGC) *in vitro* for more than three months, resulting in the increased survival of the target photoreceptors and RGC [188]. Therefore, the sustained-release drug delivery system can ameliorate ophthalmic complications by providing a stable therapeutic concentration for long durations, reducing the booster drug concentrations and additional injections necessary used in current practice. Sustained-release devices can also provide individualized treatment by combining multiple therapies, thereby tailoring to each individual needs.

## 5. Conclusion

Personalized medicine is a multi-faceted approach for physicians to individualize therapy, incorporating tailored therapeutic options and dosage optimization, as well as recent advances in genomics, proteomics and nanotechnology. It is a model for increased health systems efficiency with improved outcomes and decreased iatrogenic adverse side effects. There is a high clinical need for therapeutic personalization and dosage optimization in ophthalmology due to the sub-stratification of target patients based on pathology, as well as the need to decrease potential side effects of therapeutics. The modalities may be used in monotherapy or in combination therapy to achieve optimal results. In several retinopathies, anti-vascular endothelial growth factor therapies have been shown to enhance outcomes. There may be further personalization with different loading doses, duration of therapy and dosing frequency. In glaucoma, advances in agents that affect outflow and remodel the trabecular meshwork continue to demonstrate improved intraocular pressure control. Targeted delivery and sustained drug release are both models of dosage optimization to deliver sustained concentration of therapeutic agents without repeated invasive procedures. Regarding genomic applications to ophthalmic conditions, it will not be the cost of genotyping or sequencing that will deter the progress of personalized and predictive medicine, but rather the interpretation and clinical utility of the raw data. Instantaneous access to genotypic information for point-of-care treatment may also be a great challenge with privacy and ethical issues of pre-emptive genomic information in electronic records [189]. Biomarker technology coupled with companion clinical diagnostic laboratory tests will continue to advance medicine where customized treatment appropriate for each individual will continue to define standard of care. The level of evidence for qualifying the clinical utility of any biomarker needs to be rigorous, and the practice guidelines may continue to evolve as the field advances.
